# Assessing the Exposome with External Measures: Commentary on the State of the Science and Research Recommendations

**DOI:** 10.1146/annurev-publhealth-082516-012802

**Published:** 2017-03-20

**Authors:** Michelle C. Turner, Mark Nieuwenhuijsen, Kim Anderson, David Balshaw, Yuxia Cui, Genevieve Dunton, Jane A. Hoppin, Petros Koutrakis, Michael Jerrett

**Affiliations:** 1 Barcelona Institute for Global Health (ISGlobal), Barcelona 08003, Spain; 2 Universitat Pompeu Fabra (UPF), Barcelona 08002, Spain; 3 CIBER Epidemiología y Salud Pública (CIBERESP), Madrid 28029, Spain; 4 McLaughlin Centre for Population Health Risk Assessment, University of Ottawa, Ottawa, Ontario K1G 3Z7, Canada; 5 Department of Environmental and Molecular Toxicology, Oregon State University, Corvallis, Oregon 97331; 6 National Institute of Environmental Health Sciences, Research Triangle Park, North Carolina 27709; 7 Department of Preventive Medicine and Department of Psychology, University of Southern, California, Los Angeles, California 90033; 8 Center for Human Health and the Environment, Department of Biological Sciences, North Carolina State University, Raleigh, North Carolina 27695; 9 Department of Environmental Health, Harvard University, Boston, Massachusetts 02115; 10 Division of Environmental Health Sciences, School of Public Health, University of California, Berkeley, California 94704; 11 Department of Environmental Health Science, Fielding School of Public Health, University of California, Los Angeles, California 90095-1772

**Keywords:** exposome, external exposures, geographic information systems, remote sensing, global positioning systems, smartphones

## Abstract

The exposome comprises all environmental exposures that a person experiences from conception throughout the life course. Here we review the state of the science for assessing external exposures within the exposome. This article reviews (*a*) categories of exposures that can be assessed externally, (*b*) the current state of the science in external exposure assessment, (*c*) current tools available for external exposure assessment, and (*d*) priority research needs. We describe major scientific and technological advances that inform external assessment of the exposome, including geographic information systems; remote sensing; global positioning system and geolocation technologies; portable and personal sensing, including smartphone-based sensors and assessments; and self-reported questionnaire assessments, which increasingly rely on Internet-based platforms. We also discuss priority research needs related to methodological and technological improvement, data analysis and interpretation, data sharing, and other practical considerations, including improved assessment of exposure variability as well as exposure in multiple, critical life stages.

## INTRODUCTION

### The Exposome

The exposome, a concept first proposed in 2005, comprises all environmental exposures that a person experiences from conception throughout their entire life course (107, 108). It was intended to stimulate more comprehensive exposure assessment in epidemiology studies and investment in the development of novel exposure assessment tools and approaches, including the use of biomarker and ‘omics approaches, to support agnostic analyses of environmental influences on health. In parallel to large investments into genomic research and the broadening shift in perspective from the gene to the genome, the exposome sought to better capture highly variable exposures, both spatially and temporally, to improve our understanding of disease etiology (107, 108). The exposome can be classified into internal (e.g., metabolic processes, circulating hormones, and aging), specific external (e.g., chemical pollutants or lifestyle factors), and general external (e.g., broader socioeconomic and psychological contexts) domains, though they remain complementary and interrelated (108).

Investigators have proposed several approaches to assess the exposome. Rappaport (80) describes environmental exposures as internal biologically active chemical exposures and proposes a biomonitoring-based, agnostic approach to measuring the exposome to better understand unknown causes of human disease. In contrast, van Tongeren & Cherrie (102) describe an integrated approach to measuring the exposome by considering all available data on internal exposure, external exposure, and personal behavior, including making use of routinely collected data and data from newly developed sensors. They also note that current limitations in the measurement of internal and external environmental exposures necessitate this combined approach.

### The External Exposome

This article focuses on external exposure assessment for several reasons. Although much research using internal assessment approaches, including large-scale targeted biomonitoring (73) or untargeted metabolomics (80), has demonstrated a potential for identifying environmental health associations, these approaches also have several limitations, including the inability to identify the source, to account for the route of exposure, or to address spatial or temporal variability of exposure, each of which is critical for understanding the exposome and its link with public health protection. Also, no known or selective biomarker of current or historical exposure exists for many external exposures. There may also be complex mixtures of exposures that elicit similar health effects (e.g., noise versus air pollution). Assessment of the external environment, including broader contextual factors, is also relevant for understanding both main effects on health and also potential mechanisms of buffering or susceptibility. For example, the biological response to noise may be mediated by various individual- and contextual-level factors that affect sound perception, including innate sensitivity, coping capacity, perceptions of the source, source authorities, and general societal expectations (41). Consequently, understanding the exposome more completely must rely on distinct yet complementary information from both internal and external assessments of exposures (56, 62, 76, 90).

Here we define external exposures as those that are assessed prior to the point of entering the body (e.g., before they get under the skin). We acknowledge, however, that in some cases the distinction between internal and external domains may be unclear, such as in the case of physical activity, which may represent both a specific exposure of interest and an endogenous mediating factor (108). A related article describes assessment of the exposome in biological samples (22).

### Objectives and Conceptual Model

The objectives of this commentary are to provide an overview of (*a*) relevant categories of exposures that can be assessed externally; (*b*) the current state of the science in external exposure assessment; (*c*) current tools available for external exposure assessment; and (*d*) priority research needs in external exposure assessment in the context of the exposome.

**Figure 1:**
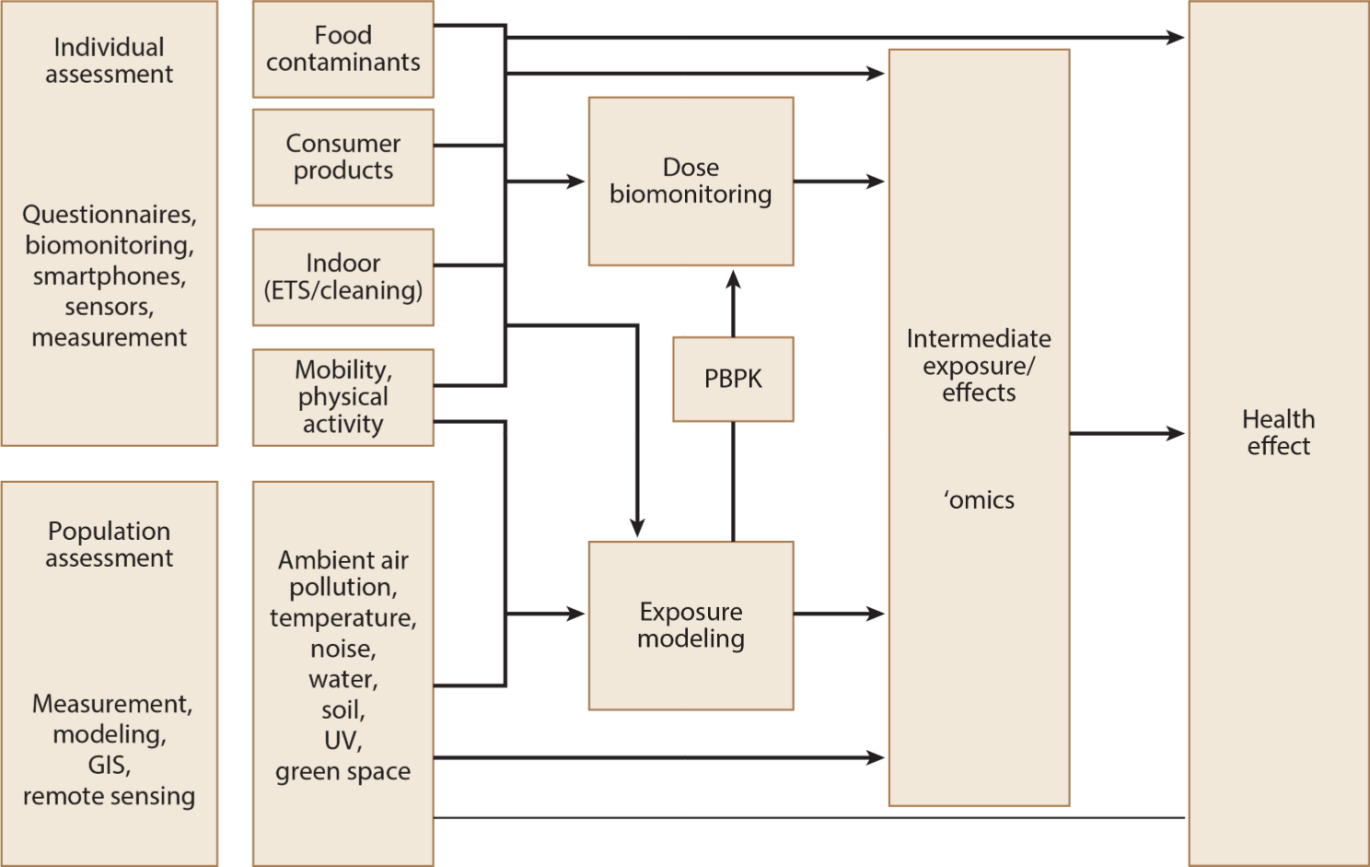
Conceptual model for the assessment of environmental exposures. Abbreviations: ETS, environmental tobacco smoke; GIS, geographic information systems; PBPK, physiologically based pharmacokinetic models; UV, ultraviolet. (Adapted from Reference 67, figure 1.5, p. 12, by permission of Oxford University Press, https://global.oup.com/academic/?lang=en&cc=us.)

This manuscript is framed in the conceptual model for the assessment of environmental exposures (67) (Figure 1). The model shows that while exposure to outdoor air pollution, temperature, noise, water and soil contaminants, ultraviolet radiation, and green space has generally been measured and/or modeled on a population level, exposure to food contaminants, consumer products, indoor pollutants (e.g., environmental tobacco smoke, cleaning products), and physical activity has generally been assessed by obtaining information from individuals. Individual assessment methods may be used to build or validate environmental models. Furthermore, information obtained from individuals (e.g., water intake, physical activity) can be combined with environmental estimates to obtain exposure estimates. Physiologically based pharmacokinetic models can be used to transform exposure into dose estimates. Environmental exposure and dose estimates can be linked with ‘omics data to obtain markers of exposure, dose, or health effect and/or to determine underlying mechanistic pathways between environment and health. Environmental exposure and dose estimates as well as ‘omics markers can also be linked to health effects to determine exposure-response relationships or their absence.

## EXTERNAL EXPOSURE ASSESSMENT

### Types of External Exposures

Table 1, developed by a National Institute of Environmental Health Sciences (NIEHS)–appointed Working Group on the state of the art of external exposure assessment, provides a listing of selected exposures that can be addressed by external exposure assessment to illustrate the broad range of exposures that may be considered. The current state of the science is described for each exposure according to various criteria, including (*a*) level of biological plausibility of potential health effects in human populations (these data may be available from existing studies or from the analogy of effects of related stressors); (*b*) known or reasonably surmised pathways of exposure (e.g., inhalation, ingestion, dermal uptake, or other pathways such as endogenous stress response); (*c*) the potential to affect large human populations or have potential large effect sizes (e.g., an attributable fraction approach); (*d*) feasibility of assessment with current or near-term new technologies on large populations; and (*e*) the capacity to measure/infer individual-level exposure or dose either through direct measurement or through models that can infer exposure on meaningful temporal and spatial scales. Here we focus on broad categories of known exposures, though we acknowledge that there may be other unknown or emerging exposures for which there is little information, including complex mixtures of pollutants. We also note that most applied studies will need to adopt either a targeted or a semitargeted approach to external assessment of the exposome to target specific exposures or groups of exposures.

For most of the exposures listed here, including outdoor air pollution and radon, for example, there is a high level of biological plausibility of health effects owing to results from previous studies and mechanistic evidence. In some cases, such as for electromagnetic fields or green spaces, the level of biological plausibility is less certain. There are also multiple known pathways of exposure for many external exposures. With the exception of individual extreme events and certain occupational exposures or infectious agents, many external exposures have the capacity to affect large populations. In contrast, for most exposures, current technology allows only a low-to-moderate feasibility of measurement on large populations, across cohorts, or population health surveys and a low-to-moderate capacity to measure/infer individual-level exposure or dose.

### State of the Science

Although traditional exposure assessment approaches have typically relied on questionnaires and static monitors (and models based on them), recent rapid technological advancement has allowed for novel assessment methods, which have generated large data sets capable of capturing exposure variability at finer scales of assessment (5). In 2005, the ad hoc Committee on Environmental Exposure Technology Development described the use of environmental sensors and geographic information systems (GIS) for deriving personalized external exposure estimates (106). We briefly review below more recent advances in external exposure assessment based on GIS; remote sensing; global positioning system and geolocation technologies; portable and personal sensing, including smartphone-based sensors and assessments; and self-reported questionnaire assessments, which increasingly rely on Internet-based platforms.

### GIS

**Figure 2:**
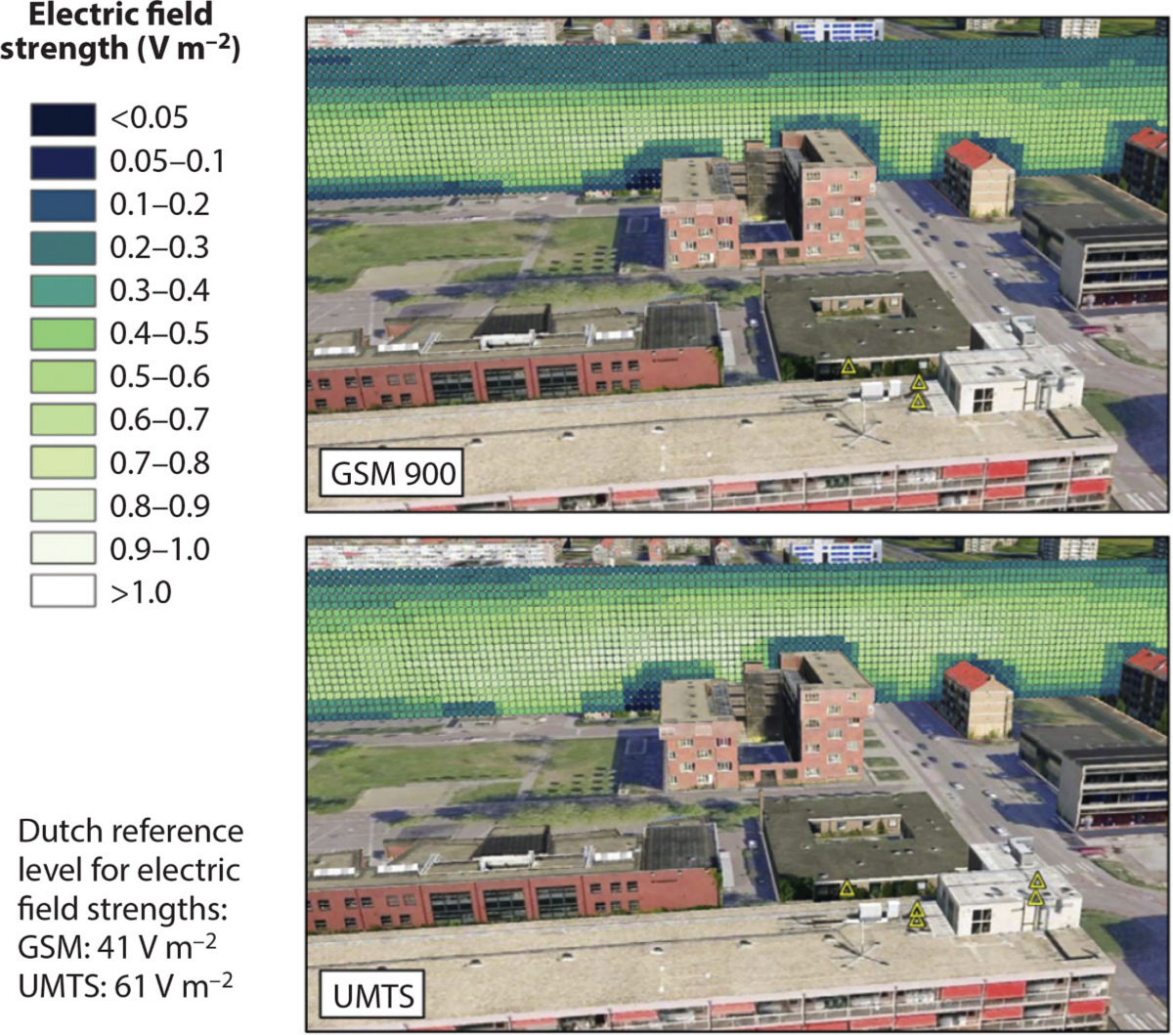
Three-dimensional profile of the Global System for Mobile Communication (GSM) (*top panel*) and Universal Mobile Telecommunications System (UMTS) (*bottom panel*) electric field strengths [volts per meter (V m^−2^)]. The yellow triangles show the locations of GSM and UMTS antennas for the top and bottom panels, respectively. Reprinted from *Sci. Total Environ.*, 445–46, Beekhuizen J, Vermeulen R, Kromhout H, Burgi A, Huss A, Geospatial modeling of electromagnetic fields from mobile phone base stations, 202–9, 2013, with permission from Elsevier.

GIS has transformed environmental health research by integrating databases that connect different attribute data by geographic location. Data on external environmental exposures obtained from remote sensing, geolocation technologies, or sophisticated modeling outputs can be combined with health attribute data obtained via personal sensing or other approaches. GIS integrates topologic geometry, which can manipulate geographic information, with automated cartography, enabling users to compile digital or hard-copy maps. GIS can quantify buffer distance between an exposure source and a human receptor and may be used to characterize proximity to roadways, factories, green spaces, water bodies, and other land uses that have either potentially adverse (e.g., ambient pesticide exposure from agricultural use) (75) or salutogenic exposures (e.g., density of healthy food stores or recreational establishments) (15). For example, NISMap, a three-dimensional GIS-based propagation model of exposure to ambient radiofrequency (RF) electromagnetic fields from cellular telephone base stations for use in epidemiological studies, has been developed to integrate building geometry and damping, topographical, and antenna/transmitter data (8) (Figure 2). GIS can also display and analyze mobility of people as they travel through the external environment.

### Remote sensing

**Figure 3:**
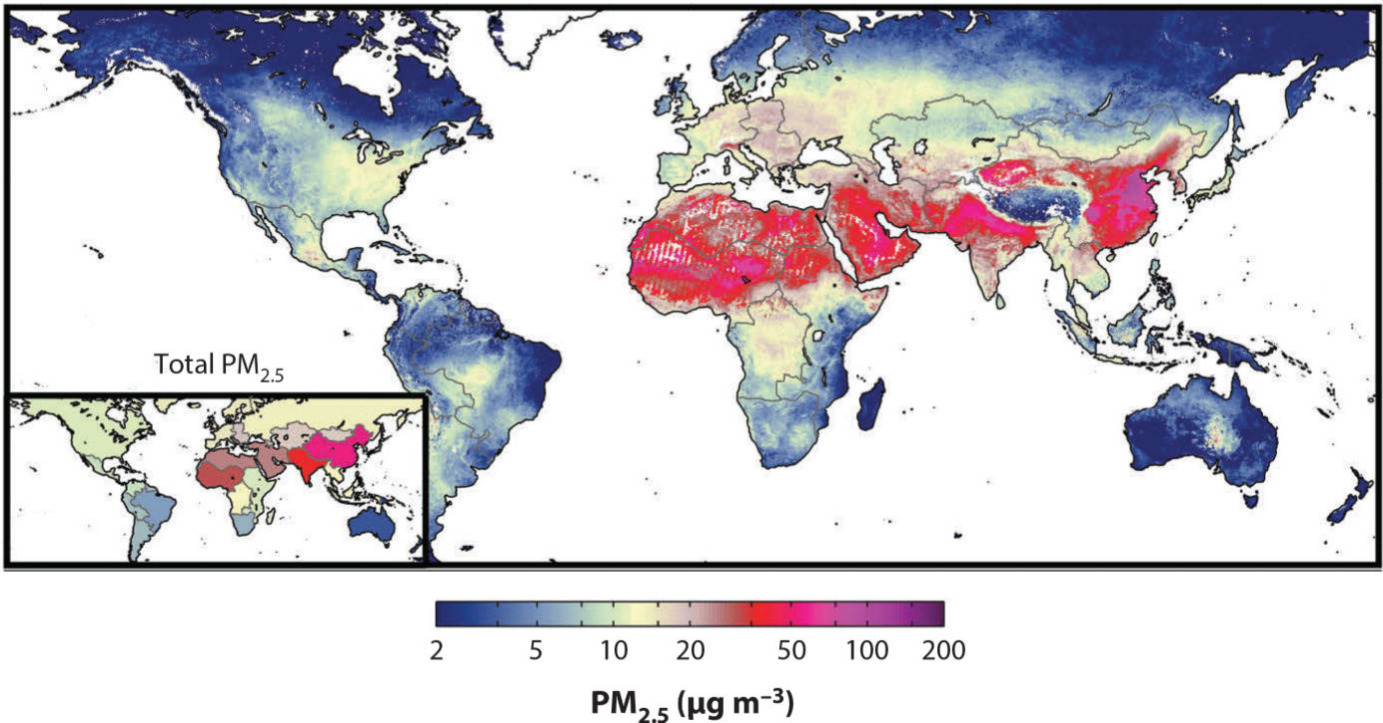
Global decadal (2001–2010) satellite-derived mean PM_2.5_ concentrations (adapted from Reference 99).

Remote sensing involves the collection and interpretation of data obtained about the surface of the earth from a distance. These technologies and related methods are useful for external exposure assessment in areas with little ground-based monitoring (42, 85). For instance, aerosol optical depth (AOD) using satellite-based technologies measures light extinction by aerosols suspended in the atmosphere in a given column and has been used in the estimation of fine particulate matter air pollution (PM_2.5_) concentrations. van Donkelaar et al. (100) estimated global PM_2.5_ concentrations using MODIS (moderate resolution imaging spectroradiometer)- and MISR (multiangle imaging spectroradiometer)-based measurements of AOD in combination with the Geos-Chem chemical transport model at a 10-km resolution. These estimates were recently improved and updated (Figure 3) with a high-level of agreement observed between satelliteand ground-based measurements in North America (*r* = 0.76), Europe (*r* = 0.73), and globally (outside North America and Europe) (*r* = 0.81) (99). Remote-sensing estimates have been used to assess associations between PM_2.5_ and cardiovascular disease in epidemiological studies (12, 14, 17). Some studies have recently used 1-km estimates of PM_2.5_ for the United States, which increase their utility for exposomics studies (55, 101). Remote-sensing techniques have also been used to estimate an expanding list of environmental exposures, including nitrogen dioxide (NO_2_) concentrations (35), green spaces (2, 70), temperature (19), the built environment (13, 95), outdoor light at night (45), agricultural chemical exposure (60), land cover classifications (11), river plumes (3), water quality (26), and marine microorganisms (39), for example.

A major advantage of remote sensing is virtual global coverage, which is promising for large population studies of the exposome. Limitations include typically broad spatial and temporal scales, which are unlikely to capture fine-level variation or short-term peak exposures. Investigators also encounter measurement limitations for PM_2.5_ in cloudy conditions, at night, or on bright surfaces (43, 99, 100). For assessment of green spaces, there is the inability to assess quality as opposed to quantity of space or to distinguish vegetation type or species (70). Jerrett et al. (49) observed stronger PM_2.5_–cardiovascular mortality associations from models that used ground-based as opposed to remote-sensing information, particularly for models that could estimate fine-scale variation from traffic sources in the United States. Hybrid approaches combining data on land use with remote-sensing estimates have been developed to downscale remote-sensing estimates horizontally (7).

### Global positioning system and geolocation technologies

The GPS allows one to track a person’s geographic position to better understand potential exposures and their contexts. The GPS has three components: a space segment with some 24 satellites that transmit signals to the earth; a control segment that tracks satellites, resets their clocks, and maintains their positions; and a user segment of individual devices that receives signals and calculates three-dimensional positions and times (38). GPS signals are also sometimes augmented by land-based navigation systems using cellular telephone triangulation (87).

Geolocation technologies have been used to improve external exposure assessment in numerous ways, including, for example, tracking potential exposure to malaria control pesticides (32), supporting infectious disease surveillance and outbreak response (34), and refining air pollution exposure estimates. GPS data can be combined with personal air pollution monitoring data, from devices carried by study subjects as they walk, ride bicycles, drive, and live their daily lives, as well as with data on physical activity and inhalation rates to allow investigators to calculate more detailed exposure estimates (47, 61, 69). Steinle et al. (93) reviewed several studies combining GPS devices, personal monitoring, and time-activity diaries to estimate personal levels of exposure to air pollutants. Data on personal levels of exposure can be used to calculate population-level exposure estimates using health and demographic information.

**Figure 4:**
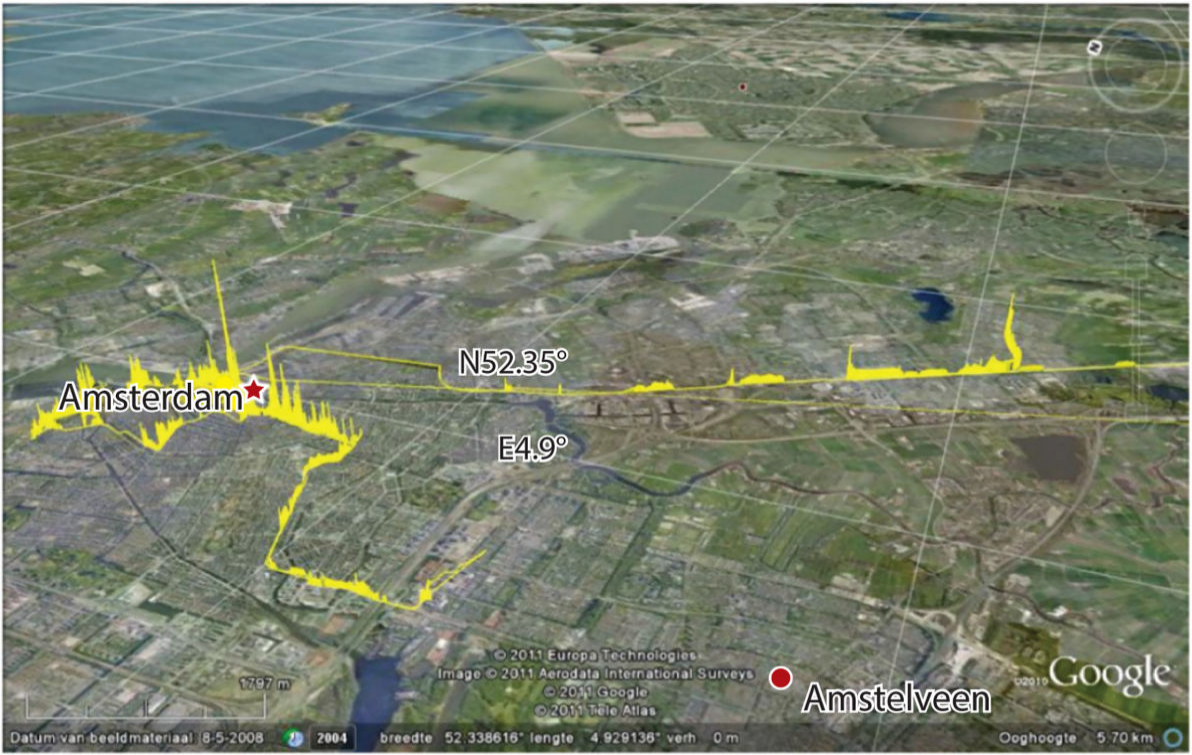
Spatial pattern of exposure (*yellow*) to electromagnetic fields from GSM base stations during travel by train, tram, and bus. The height of the yellow profile is proportional to the electric field strength (1 km represents 1 V m^−2^). Courtesy © 2011 Google, © 2011 Aerodata International Surveys, © 2011 Europa Technologies, © 2011 TeleAtlas. Reprinted from *Environ. Int.*, 48, Bolte JFB, Eikelboom T, Personal radiofrequency electromagnetic field measurements in the Netherlands: exposure level and variability for everyday activities, times of day and types of area, 113–42, 2012, with permission from Elsevier.

GPS data have also been combined with accelerometers worn on children to study how different land use configurations affect physical activity behavior (2, 48). Bolte & Eikelboom (10) assessed mean daily personal levels of RF field exposure in the Netherlands using personal monitors in conjunction with GPS-based location and time-activity data (Figure 4). Rajkovich & Larsen (78) describe a bicycle-based measurement system for thermal exposures that incorporates GPS data with measurements of air and ground surface temperature, relative humidity, solar and long-wave radiation, wind speed, barometric pressure, and sky view factor.

Geolocation technologies will likely play an increasingly integral role in widespread population-based or individualized sensing, especially with smartphone-based applications (discussed below), increasing the precision of external exposure assessment; however, limitations, including position errors indoors as well as signal interference by features of the built environment in urban settings, should be carefully considered (63). Further research into the incorporation of indoor real-time locating systems, wearable cameras, or other evolving technology to provide detailed indoor location data is needed, as is standardization of protocols for GPS data analysis (58).

### Portable and personal sensing

**Figure 5:**
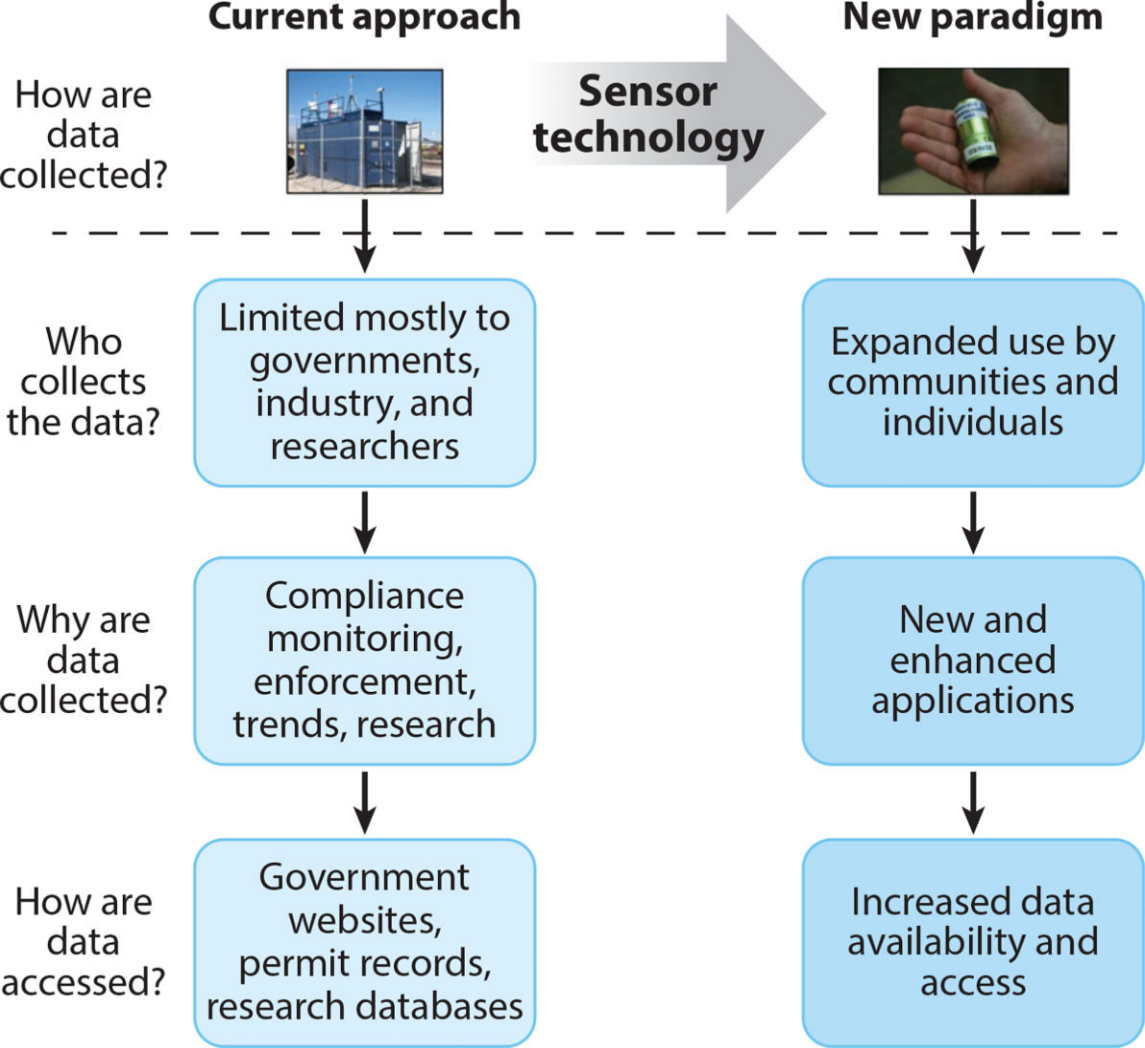
The changing paradigm of air pollution monitoring (adapted with permission from Reference 92. Copyright 2013 American Chemical Society).

A wide range of novel techniques are emerging in terms of portable and personal sensing to improve external exposure estimates and to understand patterns of population exposure. Snyder et al. (92) described the changing paradigm and recent advancements in air pollution monitoring, in particular the use of portable and often personal, low-cost, real-time sensors that offer increased spatial and temporal resolution and data availability to both researchers and individuals and communities themselves (Figure 5). Portable microsensors are increasingly being deployed by researchers and have recently been used in the development of land-use regression surfaces for NO_2_ and ozone (O_3_) in Montreal, Canada (23). Joseph et al. (50) recently described a mobile three-dimensional drone-based measurement system that can more comprehensively assess general-population RF field exposure from cellular telephone base stations.

**Figure 6:**
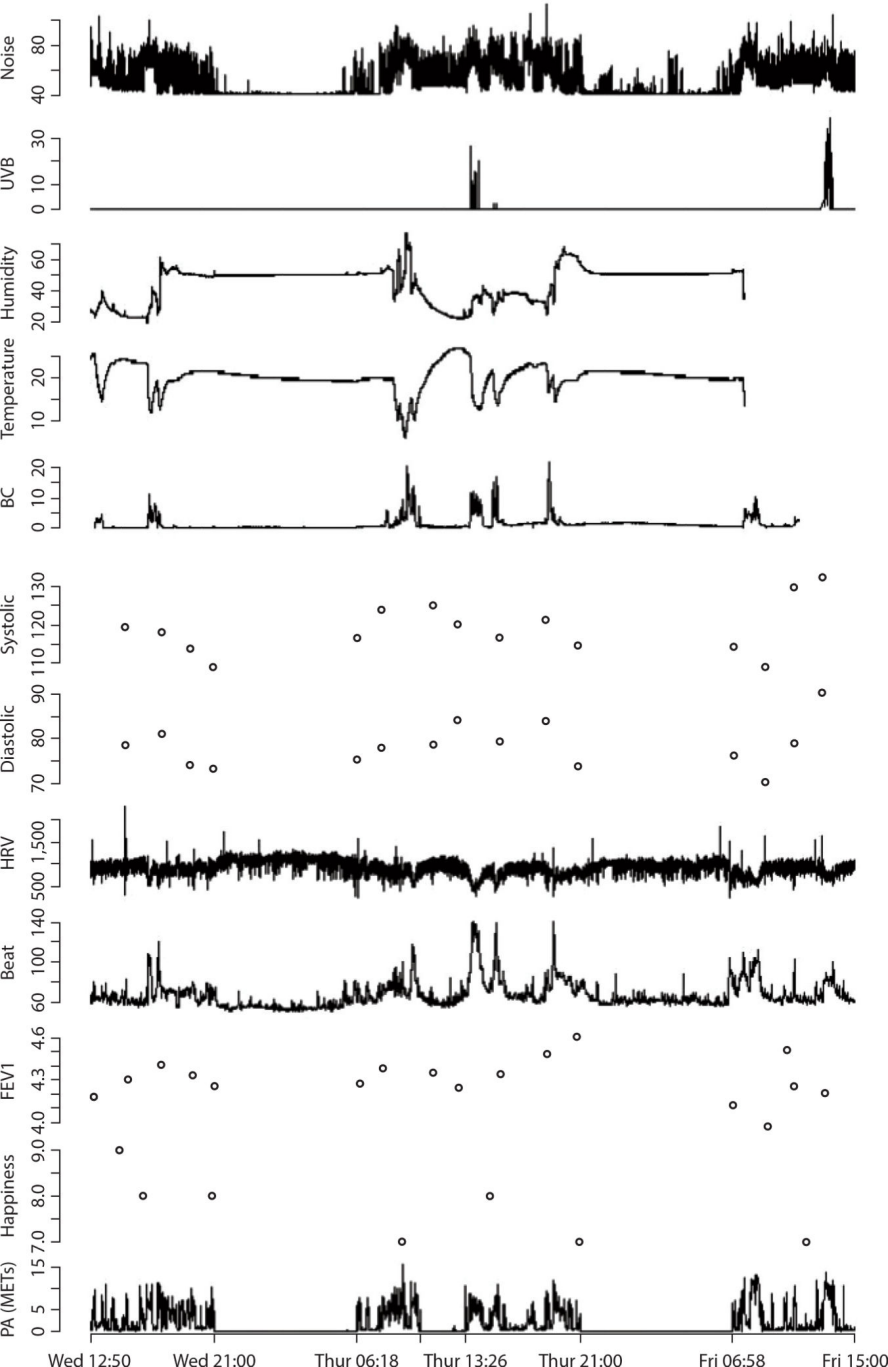
Personal levels of noise (dBA), ultraviolet B (UVB) (mJ/cm^2^), humidity (%), temperature (°C), black carbon (BC) (μg/m^3^), blood pressure (mmHg), heart rate variability (HRV) (ms), heart beat (beats per minute), lung function (L), emotional status, and physical activity (PA) [metabolic equivalents (METs) during two 24-h periods] (adapted from Reference 67).

**Figure 7:**
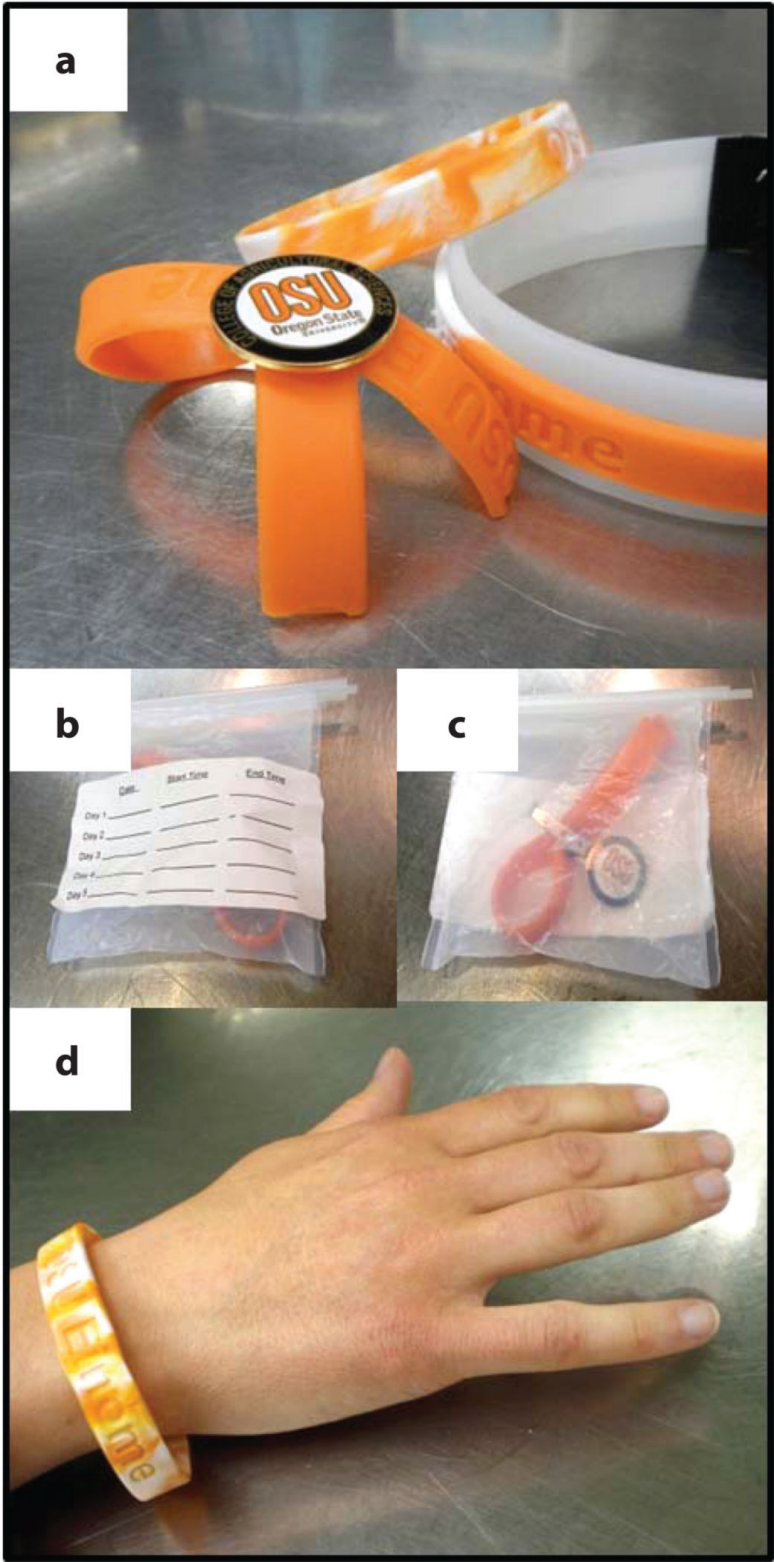
Examples of silicone personal sampling samplers. (*a*) Configurations of wristbands used in the study including a single wristband, one cut and worn as a lapel, and one worn as a stacked wristband in which only the outer band was analyzed; (*b*–*c*) bags used for transport that were attached to track participant identification and exposure time in the occupational deployments; (*d*) single wristband deployment (debossed writing as pictured: “OSU EINOME” for Oregon State University Environmental Integrated Organic Monitor of Exposure) (adapted from 71).

Nieuwenhuijsen et al. (68) reviewed advances in personal-sensing technology for external assessment of a broad range of environmental exposures, including air pollution, noise, temperature, and green space, as well as health response, including blood pressure, heart rate, lung function, emotional status, and physical activity levels (Figure 6). O’Connell et al. (71) developed a method for using silicone wristbands as inexpensive personal passive samplers for the collection of time-weighted mixed chemical exposure (Figure 7). Investigators identified a total of 49 chemical compounds out of a possible 1,182 screened following 30 days of use by public volunteers; identified chemical compounds included polycyclic aromatic hydrocarbons, consumer products, pesticides, phthalates, and various industrial compounds. This type of method offers the promise of quasi-targeted, agnostic investigations that would parallel and complement internal exposure data mining. Personal light intensity data loggers have also been used in occupational studies of night shift workers (40, 72).

### Smartphone-based sensors and assessments

**Figure 8:**
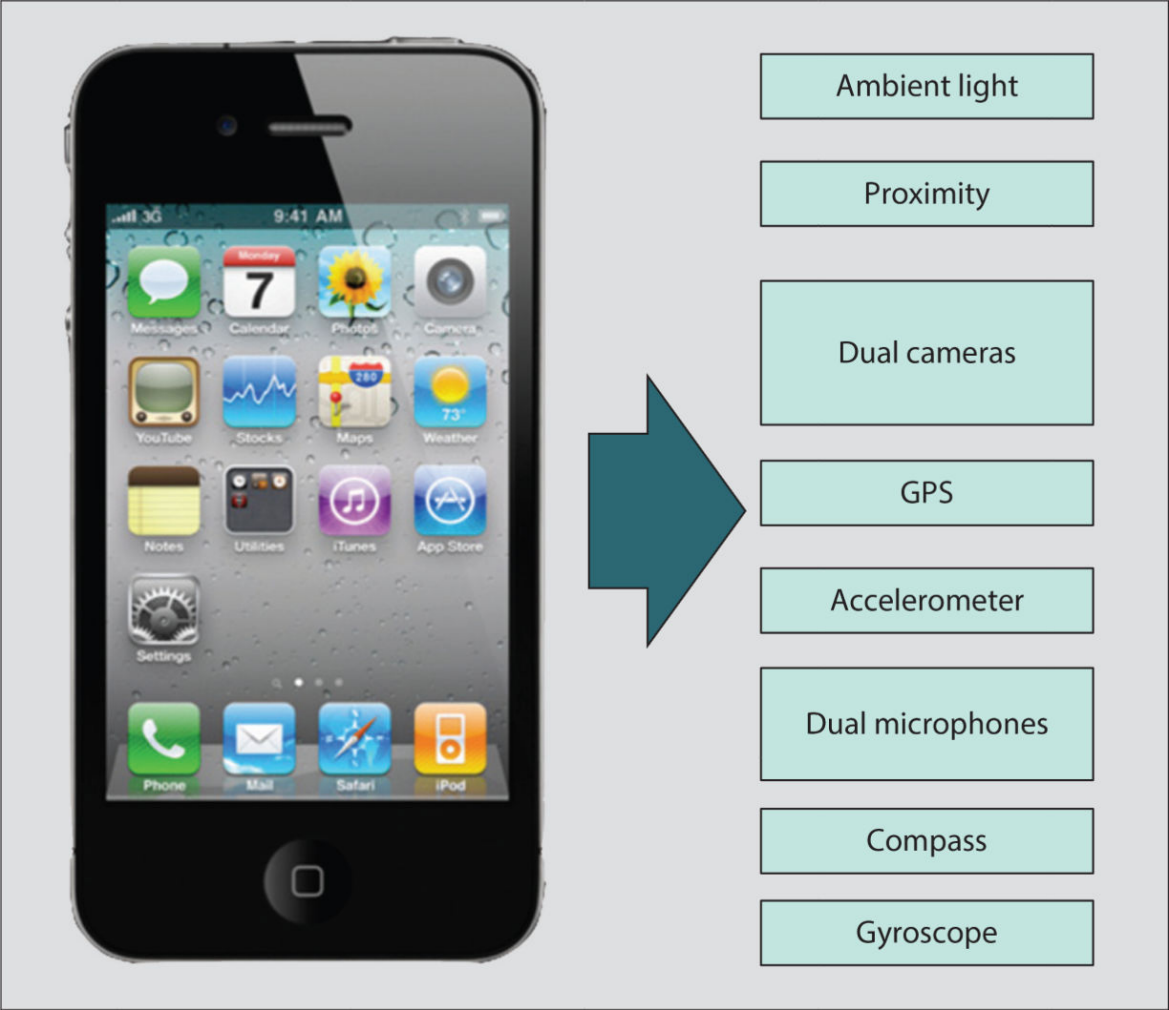
An off-the-shelf iPhone 4, representative of the growing class of sensor-enabled phones. This phone includes eight different sensors: accelerometer, global positioning system (GPS), ambient light, dual microphones, proximity sensor, dual cameras, compass, and gyroscope. © 2010 IEEE. Reprinted, with permission, from Reference 54.

Cellular telephones, carried routinely by billions of people around the world, can allow personalized monitoring of the environment as people move through time and space. Smartphones already come equipped with many embedded sensors, such as compasses, GPS, gyroscopes, accelerometers, dual cameras, dual microphones, proximity detectors, ambient light detectors, Wi-Fi, and Bluetooth connectivity that can be harnessed for personalized sensing of the external environment as well as for transmitting of data from other wearable sensors (48) (Figure 8). Ramanathan et al. (79) used a smartphone camera to photograph black carbon on a filter for processing elsewhere. Snik et al. (91) described an optical add-on, iSPEX, to measure atmospheric aerosols through spectropolarimetric measurements by citizen scientists. There was good agreement between iSEPX and spatial and temporal aerosol optical thickness as estimated from satellite- or ground-based precision photometry, respectively. Dewulf et al. (24) used routine passive mobile positioning data collected by the mobile phone network as an approach to capture individual time-location information more efficiently when estimating air pollution exposure in Belgium.

A number of software applications have been developed that exploit onboard sensors such as motion, audio (for noise), visual, and location sensors. CalFit software uses the built-in accelerometer and GPS sensors to record activity counts and energy expenditure as well as time and location information in which an activity occurs (27). Smartphone accelerometers with CalFit software performed as well as Actigraph accelerometers, the current gold standard, although wear time was considerably less for the smartphones owing to a lack of compliance by some study participants (27). Another study combined CalFit data with land-use regression estimates of NO_2_ exposure in Barcelona, Spain, and determined that transit accounted for 24% of participants’ inhaled dose of air pollution, even though it accounted for only 6% of their time (20).

Other software applications have aimed to improve our understanding of patterns of smartphone use and RF field exposure in epidemiological studies collecting data on the number of calls, call duration, laterality, hands-free device use, and communication system (36, 37). Validation studies in both young people and adults indicated that participants tended to underestimate number and overestimate the duration of calls in self-reported questionnaire assessments compared with those measured with the software application (36, 37). Investigators have explored smartphone-based noise measurement applications to address the limitations of traditional noise-mapping approaches based on prediction models (65, 86). They have also been used to administer questionnaires in a flexible time- or context-specific manner (see below).

**Figure 9:**
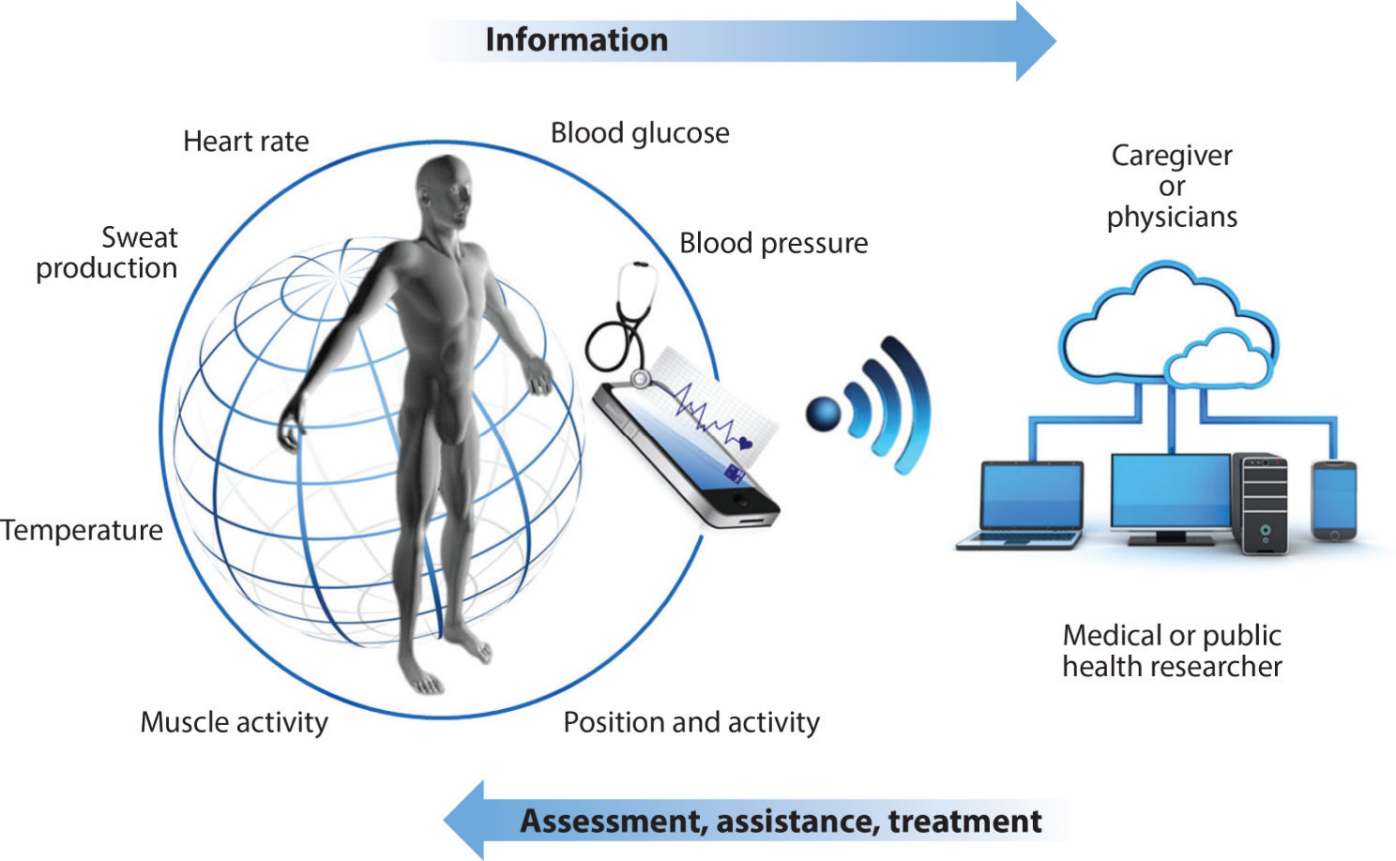
On-body sensor technology allows data collection for individual, (near) real-time, care-driven monitoring of health-related end points. Reprinted from *Int. J. Hyg. Environ. Health*, 217(8), Smolders R, de Boever P, Perspectives for environment and health research in Horizon 2020: Dark ages or golden era?, 891–96, 2014, with permission from Elsevier.

Smartphones are used in the area of mHealth (mobile health) to transmit physiological measurements and other relevant data (Figure 9). There is also increasing interest in smartwatch applications and multiple sensors for health and behavior tracking, such in the case of diabetes or Parkinson’s disease management, for example (4, 88). A growing number of telemedicine studies may expand the repertoire of possible physiological measurements that are critical to understanding biological responses to external exposures (89).

Future challenges in portable and personal sensing include measuring longer-term exposures and health outcomes, reducing cost, improving operability for application in larger population-based studies—in particular to avoid problems in compliance, potential sampling bias, and behavioral change due to wearing of the monitors—improving reliability and quality of data, measuring a greater number of exposures, and integrating and interpreting data from diverse sources. Further research to validate the expanding number of available software applications is also required (9). Future endeavors could put other devices, including miniaturized pollution monitors, into such phones.

### Self-reported questionnaire assessments

Although assessment of the exposome is based largely on objective assessments that are passively collected through sensor technology, population-based studies will still rely on questionnaires and surveys to help capture self-reported, personal characteristics and historic exposures. Questionnaires are inexpensive, effective ways to collect data from a large population. Information from questionnaires on residential and occupational history can be linked to the growing number of geospatial data sources to create integrated metrics of exposure to environmental contaminants, such as agricultural chemicals (16) or air pollution (105). Technological improvements regarding how questionnaires are administered (e.g., smartphones, social media, and social networks) have updated the utility of this commonly used tool. For example, as part of the European Physical Activity through Sustainable Transport Approaches (PASTA) project, a large-scale multicity longitudinal online survey is being conducted to better understand the determinants of physical activity and active transportation over time, including a detailed assessment of mobility patterns in daily life (28). Computer-aided questionnaires can improve the quality of participants’ reported data and allow investigators to rapidly integrate questionnaire responses into analytical data sets. Questionnaires can also capture individual perceptions of the built and physical environment such as safety, traffic, and vegetation, which may differ in substantial and meaningful ways from objective indicators. There are also opportunities for crowdsourcing of self-reported data via Web-based interfaces. For example, data on cycling safety and collisions can be collected online by a global mapping system (66).

Data on perceptual information can also be gathered through context-sensitive ecological momentary assessment (CS-EMA) through real-time self-reported smartphone assessments. The system can, for example, request a person to respond to a survey either at random or when particular events are sensed through a smartphone-enabled system, such as the use of steroid inhalants (31), a period of physical activity (29), contact with nature (25), or air pollution exposures. EMA surveys provide rich data on mood, stress, social context, environmental perceptions, or behaviors at the point of contact between the exposure and receptor (30, 46). However, due to their greater frequency, EMA measures have the potential to be burdensome for participants.

Questionnaires will remain a key tool for external exposure assessment, given their low cost, ease of administration, and ability to capture perception data. Although most questionnaire measures do not capture exposure to specific compounds, many questionnaire-based metrics have been standardized and applied internationally and have proven predictive value in health assessments. Future developments will focus on mode of delivery and interaction between participants and smartphones or other devices to tailor data collection for key time windows of exposure.

## CONCLUSIONS

Despite numerous advances in external assessment of the exposome, there are a number of priority research needs related to methodological and technological improvements, data analysis and interpretation, data sharing, and other practical considerations. Research recommendations related to internal exposure assessment of the exposome, biological impact, epidemiology, and informatics and data analytics are provided in related manuscripts (18, 21, 22, 59, 94).

### Methodological and Technological Improvements

Major initiatives for methodological and technological improvements include the conduct of repeated population censuses of exposure, increased involvement of citizen scientists, and the development and validation of technologies for measurement of multiple priority analytes.

Repeated population censuses of external exposures could be based in either new or existing large-scale cohort studies or conducted cross-sectionally. Although longitudinal collection, such as in the Human Early-Life Exposome (HELIX) study, which seeks to assess pre- and postnatal external environmental exposures in existing European birth cohort studies (104), allows researchers to examine associations with health outcomes over time, a cross-sectional approach, embedded in ongoing population health surveys, such as the National Health and Nutrition Examination Survey (NHANES), would also provide useful data on population-level exposures and could be combined with data from smaller cohorts. Additional sensors and technologies could be added as part of the data collection protocol and would help provide data on spatial and temporal trends in exposure and could be used to inform future studies. The NHANES, for example, has already deployed accelerometers for measuring physical activity, and these initiatives could yield rich information from a survey that has already been used for internal exposomics inquiries (33).

There is also increasing interest in citizen science approaches to external exposure assessment, which seek to engage and empower the public in data collection efforts and prevention applications (53, 57). For example, the public health exposome concept seeks to further community engagement in health disparities research through the use of public participatory GIS to provide communities access to infrastructure to support research and decision making (51). Examples of other citizen science initiatives include community monitoring of PM_2.5_ in the Imperial Valley of California as part of a collaborative effort of the advocacy group Comite Civico del Valle, the California Department of Public Health, and several universities (http://www.ivan-imperial.org), as well as the European Citi-Sense project that is working in several countries to empower citizen volunteers to use various technologies to assist in understanding the risks they face from environmental exposures and to improve their local environmental conditions (http://www.citi-sense.eu).

Improved technologies for the measurement of multiple priority analytes are also needed, particularly those that are low-cost and applicable in large-scale studies, including new portable and personal sensors with improved measurement duration or remote-sensing technologies at finer levels of spatial resolution. There is also a need for improved assessment of exposure variability, including minute, daily, and yearly variability, as well as peak and intermittent exposures, in multiple, critical life stages, including the targeted development of standardized external exposure metrics for use in utero, in early childhood, in adolescence, and in senescence (82, 108). Analytical platforms based on high-resolution mass spectrometry have also been applied in quantitative and qualitative analysis of contaminants in various exposure matrices such as surface water and house dust (77, 84). Coupling with different extraction and separation techniques, these highly sensitive analytical platforms not only enable quantitation of targeted contaminants but also allow for suspect screening and nontargeted analysis of environmental exposures based on how the data are processed (1). Data processing is currently still a major hurdle for scaling up the application of untargeted analysis in exposure assessment, including identification of unknown compounds.

Key here is close partnership between researchers, the government, and the industry to develop useful technology that is also economical for research purposes. For example, there is increasing interest in the use of data from social media networks, particularly georeferenced data and omnidirectional imagery (e.g., Google Street View), in assessing the social and built environment (83). There may also be opportunities to build on recent developments in the fields of eHealth and mHealth, including biological sensing, and real-time patient monitoring, including additional opportunities for measurement validation. Although in some cases the development of such technologies will require smaller-scale studies with detailed validation protocols, investigators will eventually need to consider deploying these tools in larger studies. Further development of methods for predictive modeling of external exposures to both the individual and populations is also needed.

### Data Analysis and Interpretation

External exposure assessment in exposome studies involves large amounts of data collected at multiple scales and life stages. Through untargeted exposure assessment and studies of mixtures and different exposure routes, we know that humans are exposed to numerous potentially toxic chemicals. Major challenges include how to integrate and interpret data in a meaningful way, how to account for shared exposures, how to integrate data across multiple spatial and temporal scales and methodological approaches, and how to account for measurement errors.

The aggregate exposure pathway (AEP) framework, a conceptual framework that complements the adverse outcome pathways (AOP) concept, organizes exposure and toxicological data from source to dose and to outcome (97). Together, the two frameworks complete the view of the exposure–outcome continuum to enable knowledge integration and better understanding of the health impacts of chemical exposure. In addition, the AEP framework supports exposure modeling and exposure forecasting by organizing exposure data within individual units of prediction that are common to the field.

Few studies have attempted to comprehensively quantify correlations between multiple exposures in exposome studies. In an analysis of 81 environmental exposures assessed during pregnancy via a range of biomonitoring, geospatial modeling, remote sensing, and questionnaire approaches, Robinson et al. (81) reported a weak correlation (median correlation = 0.06) between exposures overall but a stronger correlation (median correlation = 0.45) between exposures within the same family (e.g., noise, water, or air pollutants), which suggests that adjustment for potential confounding between families of exposure may be permitted in future epidemiological studies of the exposome. The authors also note that correlations may be inflated for exposures assessed using a similar methodological approach, e.g., the same analytic platform or modeling input variables, possibly obscuring true exposure variability. Patel & Manrai (74) constructed an “exposome globe” to identify and display correlated clusters of exposures by extending unsupervised learning approaches originally developed for use with genomic data to 81,937 environmental exposures collected as part of four consecutive NHANES surveys in the United States. Results of these and related future studies will help us better understand routes of exposure, interpret effect estimates, appropriately identify and adjust for potential confounding, and support collaborative research efforts of related exposures (74).

Owing to rapidly evolving technology and limitations inherent in individual approaches to external exposures assessment, methods will also need to be developed to integrate external exposure data assessed across multiple spatial and temporal scales and approaches (e.g., the fusion of remote sensing with ground-based air pollution data). Statistical methods will need to account for measurement errors that may occur across scales of measurement with different measurement precision and analyte. For example, Hoffmann et al. (44) recently used a Bayesian hierarchical approach to modeling uncertainties in retrospective and prospective radon exposure assessment in a study of lung cancer in uranium miners. Furthermore, Zidek et al. (110) established that with two predictor variables in a regression model, the one that is measured with more precision will likely dominate, even if the variable measured with less precision has a stronger underlying relationship with the outcome. The potential for this kind of error to lead to false discovery increases in the presence of multiple exposures that will likely be measured with different levels of precision in exposome studies. Other unique biases such as technology-related participation biases might also occur through the use of multiple measurement tools with different sampling strategies (108). Additional research to further develop approaches to capture time-varying effects, bidirectionalty, intraindividual variability, idiographic effects, reciprocal relationships, and feedback loops is also required.

### Data Sharing

In light of the large quantity of data on the external exposome that may be generated through both individual studies and population censuses of exposure, as well as the large-scale transdisciplinary consortiums involved, an information exchange resource/clearinghouse to facilitate the sharing of exposure data, exposure assessment tools, and modeling methods from multiple studies is needed.

Such a data-sharing resource may follow the approach of currently available platforms such as Tox21 (http://ntp.niehs.nih.gov/results/hts/index.html) or the National Center for Health Statistics (NCHS, http://www.cdc.gov/nchs/), particularly for high-priority exposures. The ISA-TAB-Nano specification allows for the sharing of nanomaterial data in a spreadsheet-based format across data resources (98). Key here is the development of standardized data collection or modeling protocols as well as protocols for data annotation, structure, sharing, and use to allow for both current uses and comprehensive analyses of exposure across populations in the future (6). As another example, detailed genotypic and phenotypic data from the large UK Biobank prospective study is available as an open access online resource for researchers (96).

Careful consideration regarding privacy concerns and access to data is required because detailed geolocation data and other personal data may be collected, including social contacts and individual behaviors in some studies. In addition, particularly in exposome studies, real-time personal-level data on external exposures may be captured, and protocols for sharing the data with participants may be required because the possibility for individual-level intervention, such as exposure warnings, exists (83). Further research on risk communication with study participants may be useful (64). We emphasize, however, that the goal of exposome studies is to better understand disease etiology and environmental risk factors at the population level rather than at the individual level and that participant privacy should be protected while also enabling the potential benefits of the data to be realized (108).

### Practical Considerations

We must also evaluate the practical considerations related to operational parameters, training, and funding, including balancing costs versus necessary accuracy for technological deployment in large-scale studies. We also need relevant educational and outreach opportunities to provide adequate training to current and future researchers and research users to facilitate transdisciplinary collaborations on both targeted and broad-spectrum external exposure applications (62). There are also funding implications, such as the need for larger exposome-related research grants and transdisciplinary research centers, though this challenge does not preclude the use or leveraging of existing resources, including incentives for multisector (public and private sectors) initiatives to integrate the exposome into ongoing work. Notably, the NIEHS recently launched a competitive funding infrastructure to support exposome-related research for children’s health [Children’s Health Exposure Analysis Resource (CHEAR)], including a laboratory network, a coordinating center, and a data center to facilitate opportunities for data integration and pooled analysis of a broad range of environmental exposures, including lifestyle and social environment exposures (https://www.niehs.nih.gov/research/supported/exposure/chear/).

In conclusion, although many priority research needs and challenges related to external exposure assessment of the exposome remain, it is important to begin to conduct such work because much can be learned from practical research experience that uses a coordinated and thoughtful approach. For example, existing databases may be able to examine priority stressors that are of interest in the short term, which should be identified [i.e., Expocast (http://www.epa.gov/ncct/expocast/) or the Toxin-Toxin-Target Database (T3DB) (http://www.t3db.ca)] (109). Much insight can also be gained from three large initiatives funded by the European Union, which are investigating the feasibility and utility of assessing the exposome [e.g., EXPOsOMICS (103), HELIX (104), and Health and Environment-Wide Associations based on Large population Surveys (HEALS) (http://www.heals-eu.eu/index.php/project/)]. These European studies of the exposome, as well as the ongoing Japan Environment and Children’s Study (JECS) (52), are focusing on improved measurements of known exposures (and related molecular profiles) as a first proof-of-concept approach.

While still formative, these studies promise to assess the feasibility of many new methods of exposure assessment, discovery analysis, and data integration. There are currently no ongoing studies in other continents, and continental-scale initiatives will be needed to assess the feasibility of exposomics approaches in North America and beyond. Existing and future large-scale initiatives promise to test the validity of external exposure assessment in ways that smaller studies will undoubtedly miss, particularly with respect to sensing multiple analytes in large populations.

## Figures and Tables

**Table 1:** Selected external exposures categorized according to level of biological plausibility, known or surmised pathways of exposure, capacity to affect large populations, feasibility of conducting external measurements on large populations, and capacity to measure/infer individual-level exposure or dose

Exposure	1. Biological plausibility	2. Known or surmised pathways of exposure	3. Capacity to affect large populations	4. Feasibility of conducting external measurements on large populations	5. Capacity to measure/infer individual-level exposure or dose

**ENVIRONMENTAL**					

**Air**					

Outdoor air pollution (human origin)	H	H (I)	H	L	M

Indoor air pollution (human origin)	H	H (I)	H	L	M

Pollen/mold/fungus (natural origin with human influence)	H	H (I, In, D, Mu)	H	L	L

Dust (phthalates/metals/pesticide residues)	H	H (I, In, D, Mu)	H	L	M

Noise	H	H (P)	H	M	M

**Radiation**					

Radon	H	H (I)	H	L	M

Ultraviolet radiation	H	H (P)	H	L	M

Electromagnetic fields	M	H (P)	H	M	M

**Water**					

Surface water contamination (infectious agents/chemicals)	H	H (In, D, Mu)	H	L	M

Groundwater contamination (infectious agents/chemicals)	H	H (In)	H	M	M

Drinking water (chlorination by-products)	H	H (I, In)	H	L	M

**Weather**					

Heat/humidity	H	H (P)	H	H	H/M

Extreme events (e.g., lightning-induced asthma from pollen release)	M	H (Mu)	M	M	M

**Consumer products**					

Flame retardants (PBDEs)	M	M	H	L	L

Fragrance products (musk, musk ketone)	M	L	M	L	L

Nicotine products	M	H	L	M	M

Flea products (fipronil)	M	M	L	L	L

**Built environment**					

Ambient light	M	M (P)	H	L	M

Green/blue space	M	H (Mu)	H	M	M

Point, line, and area sources that emit numerous exposures (e.g., oil refineries, roadways, ports, goods movement, emergencies)	H	M	H	H	L, depending on specific exposure

**Other**					

Soil contamination	H	H (In, In, D, Mu)	H	L	M

**OCCUPATIONAL**					

Volatile organic compounds (benzene, naphthalene)	L–H, depending on specific occupation	H (Mu)	L–H, depending on prevalence	M	M

Phthalates (diethyl phthalate-fragrance vehicle, butyl benzyl phthalate-plasticizer for floor tile, carpet backing)	L–H, depending on specific occupation	H (Mu)	L–H, depending on prevalence	L	L

Polycyclic aromatic hydrocarbons (retene, phenanthracne, dibenzo[a]pyrene)	L–H, depending on specific occupation	H (Mu)	L–H, depending on prevalence	M	L

**LIFESTYLE**					

Smoking	H	H (I)	M–H, depending on prevalence	M	L

Physical activity	H	H (P)	H	M	M

Diet	H	H (In, D)	H	L	L

Drug abuse	H	H (In, P)	H	L	L

Alcohol abuse	H	H (In)	H	M	L

**SOCIAL**					

Violence, crime, social disorder, inequality, racism, discrimination, hate crimes, etc.	L–H, depending on specific stressor	M (P)	H	L	L–M

Stress	M	M (P)	H	L	M

**INFECTIOUS AGENTS/VECTORS**					

Birds, pigs, rats, bats, other species capable of causing health effects or increasing susceptibility	L–H, depending on specific stressor	M (P, In, I, Mu)	L–H, depending on prevalence	L	L–M

Abbreviations: D, dermal absorption; H, high; I, inhalation, In, ingestion; L, low; M, medium; Mu, multiple (i.e., more than two); O, other; P, physical; PDBE, polybrominated diphenyl ether.
